# Polyoxometalates in Oxidative Delignification of Chemical Pulps: Effect on Lignin

**DOI:** 10.3390/ma3031888

**Published:** 2010-03-16

**Authors:** Biljana Bujanovic, Sally Ralph, Richard Reiner, Kolby Hirth, Rajai Atalla

**Affiliations:** 1 SUNY-ESF, Department of Paper and Bioprocess Engineering, Syracuse NY, 13210, USA; 2 USDA Forest Service, Forest Products Laboratory, Madison, WI, USA; E-Mails: sralph@fs.fed.us (S.R.); rreiner@fs.fed.us (R.R); khirth@fs.fed.us (K.H.); 3 Cellulose Science International, Madison, WI, USA; E-Mail: rhatalla@celscint.com (R.A.)

**Keywords:** polyoxometalates, keggin type, lignin, delignification, aerobic, anaerobic, lignin reactions, products

## Abstract

Chemical pulps are produced by chemical delignification of lignocelluloses such as wood or annual non-woody plants. After pulping (e.g., kraft pulping), the remaining lignin is removed by bleaching to produce a high quality, bright paper. The goal of bleaching is to remove lignin from the pulp without a negative effect on the cellulose; for this reason, delignification should be performed in a highly selective manner. New environmentally-friendly alternatives to conventional chlorine-based bleaching technologies (e.g., oxygen, ozone, or peroxide bleaching) have been suggested or implemented. In an attempt to find inorganic agents that mimic the action of highly selective lignin-degrading enzymes and that can be applicable in industrial conditions, the researchers have focused on polyoxometalates (POMs), used either as regenerable redox reagents (in anaerobic conditions) or as catalysts (in aerobic conditions) of oxidative delignification. The aim of this paper is to review the basic concepts of POM delignification in these two processes.

## 1. Lignin

**Figure 1:**
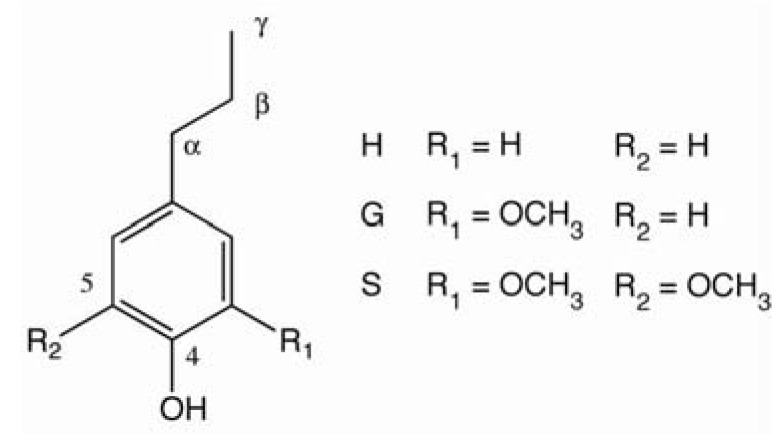
Lignin phenylpropanoid units.

Lignin is an integral cell wall constituent in all vascular plants, including herbaceous varieties. Lignin provides rigidity, water-impermeability, and resistance against microbial attack. Its amount in lignified plants ranges from 15 to 36% by mass. Lignin is an aromatic polymer consisting of guaiacyl‑ (G), syringyl- (S), and *p*-hydroxyphenyl- (H) phenylpropanoid units (Figure 1), whose proportions differ with the botanical origin of the lignin. 

**Figure 2:**
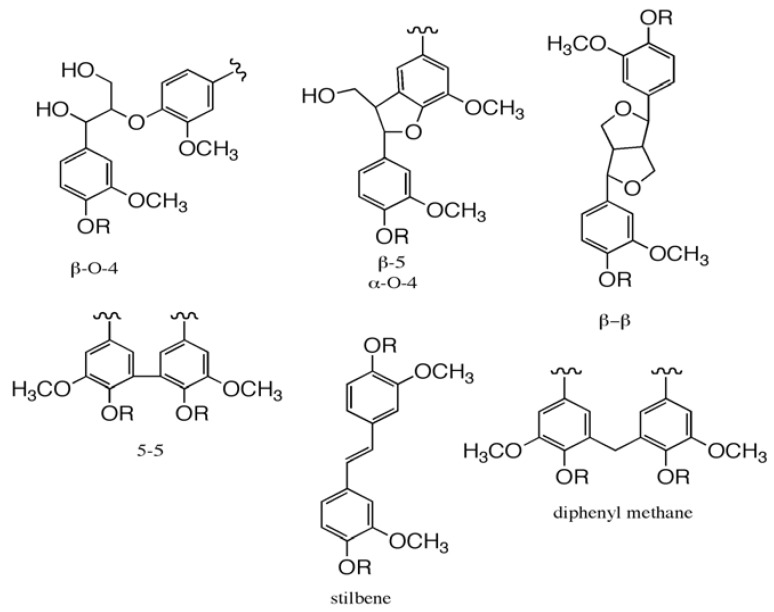
Illustrations of typical softwood lignin structures; β-O-4, β-5, α-O-4, β-β and 5-5, additionally, for residual lignin stilbene and diphenylmethane structures. R=H (non-etherified structure); R = Lignin (etherified structure).

The phenylpropanoid units are attached to each other by a series of C-O-C and C-C bonds such as β-O-4, β-5, α-O-4, β-β, and 5-5 (Figure 2). The polymer is branched, and crosslinking occurs. Lignin polymer models are well illustrated elsewhere [2]. A small percentage of the lignin building blocks are not derived from the traditional monolignols, the hydroxy cinnamyl alcohols; these include dihydroconiferyl alcohol units, as well as units with aryl conjugated carbonyl/carboxyl groups (e.g., vanillin, cinnamaldehydes: conifer- and sinapaldehyde; vanillic acid, and the cinnamic acids: ferulic and sinapic acid). Some lignins are acylated with *p*-hydroxybenzoate (poplar species) or with *p*-coumarate (grasses) [1]. The lignin in the cell walls is intimately mixed with the polysaccharides, and there are indications of the occurrence of linkages between lignin and carbohydrates (lignin carbohydrate complex, LCC). Among the proposed chemical linkages, the benzyl ether and ester types, whose formation is associated with quinone methide rearomatization reactions during lignin biosynthesis, have been considered the most probable [3].

## 2. Pulping and Bleaching

In the pulp and paper industry, lignin is removed chemically in pulping processes such as the kraft process. Conventional kraft cooking is performed in an aqueous solution of delignification agents, NaOH and Na_2_S, at the cooking temperature of 155–175 °C for a period dependent on the raw material. During this process, lignin is degraded into smaller alkali-soluble fragments, mainly through the cleavage of α- and β-aryl ether bonds. The rate of delignification decreases significantly when ~90% of the lignin has been removed [4], most likely due to the accumulation of the alkali stable bonds within lignin and between lignin and polysaccharides/hemicelluloses [5]. These recalcitrant structures may be inherently present in wood; biphenyl (5–5), or they may be formed during pulping; diphenylmethane, stilbene (Figure 2) [6,7,8]. 

Because the selectivity of delignification at this stage is severely reduced, the process is stopped, and the remaining or residual lignin is removed using bleaching agents. Removal of the residual lignin from kraft pulp can be performed in a bleaching sequence consisting of chlorination (elemental chlorine (C), or chlorine dioxide (D)) and alkaline extraction (E) steps such as CED or CEDED [4]. However**, **as environmental regulations have became more restrictive regarding the discharge of chlorinated organic compounds into the environment, the pulp and paper industry has been forced to reduce the use of chlorine-containing bleaching reagents. In one approach, conventional chlorine-based bleaching agents have been partially replaced by oxygen used as a bleaching agent (O). About one-half of the residual lignin can be removed from kraft pulp without significant deterioration of pulp quality, *i.e.* at high selectivity, by applying oxygen in an alkaline medium [4]. 

Since oxygen is a readily available, environmentally-friendly chemical of low cost, its expanded use would be advantageous. Currently however, due to the limited selectivity of oxygen, high brightness pulps are produced by applying different sequences with an initial oxygen stage; for example, oxygen followed by peroxide (P) with a chelating stage (Q) in between (OQP) [9]. Various other chlorine-free agents have been studied or implemented for use in bleaching of kraft pulps. Among these are: chemical agents (ozone (Z) and peracids (peracetic acid)); and biological agents (enzymes (laccase-mediator systems)) [10,11]. Despite many advantages, totally-chlorine-free (TCF) bleaching processes based on these agents require further improvement. For example, methods to avoid the undesirable degradation of polysaccharides by a number of reduced oxygen species formed via radical-chain autooxidation processes (superoxide radical anions, OO^.-^; hydroxyl radicals, HO^.^) should be developed [10]. In enzyme bleaching, there is a need for the development of efficient mediators [11]. In addition, TCF bleaching agents such as ozone and laccase-mediator systems tend to be of higher cost [11,12].

Polyoxometalates (POMs) favorably embrace the advantages of both chemical (active at elevated temperatures) and biological (highly selective) lignin-oxidizing agents. They have been under investigation as promising alternative bleaching agents, which may provide a basis for closed-mill delignification technologies. These studies have included the application of POMs as regenerable redox reagents [13,14] or as catalysts in oxygen bleaching [15,16].

## 3. Polyoxometalates in Bleaching

**Figure 3:**
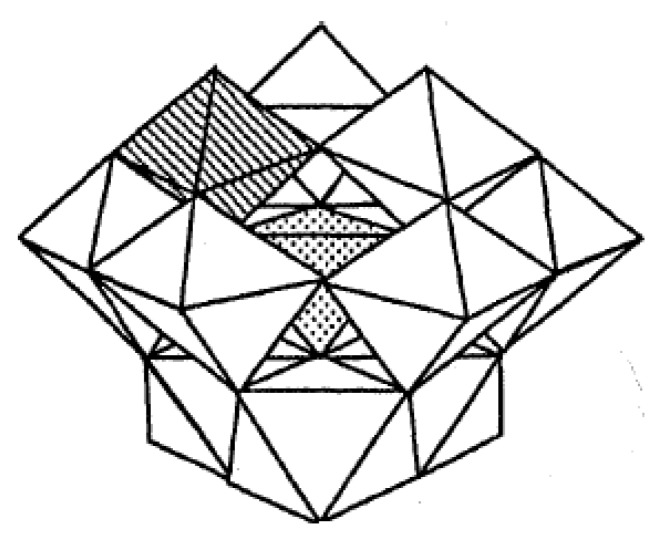
Polyhedral representation of monosubstituted Keggin-type POM [X^n+^VW_11_O_40_]^(8-n)-^ where the central tetrahedron represents the XO_4_(X^n+^ = Al^3+^, Si^4+^ or P^5+^) unit, the shaded octahedron represents the VO_6 _unit and the unshaded octahedral represent the WO_6 _units.

Among a large number of varieties, the Keggin–type polyoxometalates have been recognized as the most suitable POMs for use in oxidative delignification. The advantages of Keggin–type polyoxometalates include a range of redox potentials, solubility and molecular charges which can be adjusted during synthesis, and a relatively easy regeneration by oxygen, hydrogen peroxide, or ozone. Keggin-type heteropolyoxoanions are described by the general formula XM’_a_M”_12-a_O_b_^m-^, where X^n+^ is a d- or p-block “heteroatom” (X^n+^ = Al^3+^, Si^4+^, P^5+^), and M’ and M” are d^n^ and d^0^ metal centers, respectively (Figure 3). 

The Keggin structure has a diameter similar to that of phenylpropanoid units in lignin (~1.1nm). The negative charge of POMs anions can be counterbalanced by hydrophilic cations (e.g., Li^+^, Na^+^, K^+^, NH_4_^+^) which provide for the solubility of the resulting complexes in water; or by hydrophobic cations (e.g., Ph_4_P^+^, Et_4_N^+^), which provide for solubility in organic solvents [13,17].

The concept of delignification with POMs, as robust inorganic systems which provide controlled environments with transition-metal ions, has been developed to mimic the action of fragile lignin peroxidases containing iron protoporphyrin IX, in selective oxidation of lignin. POMs are reduced while lignin is oxidized (reaction 1); POMs may be re-oxidized by oxygen, hydrogen peroxide, or ozone (Ox = O, P, Z) (reaction 2).

Lignin + POM_ox_ → Lignin_ox_ + POM_red_ (1)

POM_red_ + Ox → POM_ox_ + H_2_O (2)

POMs, with a reduction potential sufficiently high to oxidize lignin but low enough for reoxidation with oxygen, are capable of transferring electrons from lignin to oxygen (E^0^_lignin _< E^0^_POM _< E^0^_O_), and act similarly to lignin peroxidases in white-rot Basidiomycetes fungi. The redox potential of phenolic lignin units varies from +0.45 to 0.69 V (*vs.* NHE (normal hydrogen electrode), pH 2), while the redox potential of O, Z, and P is +1.23, +2.07, and 1.76 V (*vs.* NHE, pH 1.0), respectively [18,19]. In accordance with these criteria, POMs with a redox potential in the range of +0.7 to 0.8 V *vs.* NHE have been used for delignification [13,20]. In delignification performed under anaerobic conditions, lignin is oxidized by POMs (reaction 1) in one stage and reduced POMs are re-oxidized (reaction 2) by oxygen at elevated temperatures in a separate stage; that is, the two reactions of transferring electrons from lignin to oxygen are performed in different process stages [13,14]. In an aerobic process, by contrast, lignin is oxidized (reaction 1) and reduced POMs are re-oxidized (reaction 2) in one stage [16,20]. Because POMs are recycled and can be repeatedly used in a closed system, both of these two systems are considered promising alternatives to chlorine-based bleaching systems and a step towards effluent-free bleaching plants. However, each system has its own advantages and disadvantages. Both systems will be described in the following sections.

### 3.1. Delignification with POMs: Anaerobic Conditions

**Figure 4:**
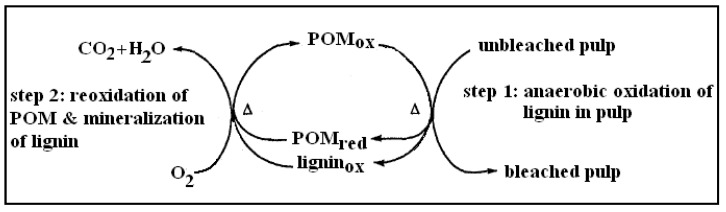
Two steps in POM bleaching.

The concept of delignification based on POMs used as lignin oxidants was developed in the mid 1990’s [21]. The two steps in which this process occurs are summarized in Figure 4.

In the first step, performed under anaerobic conditions at elevated temperature (100–140 °C), lignin is oxidized and dissolved in an aqueous solution of POMs, which are simultaneously reduced. After being treated with POMs, the pulp is separated from the spent POM liquor and washed. It has been demonstrated that polyoxometalates are readily washed from the pulp with high washing efficiency of 99.9% and greater [13] as the size of the Keggin anion corresponds approximately to the height of a phenylpropanoid unit (°11 Å) and because cellulose is also negatively charged [22,23]. It is expected that the industrial application would require an advanced industrial washer, such as a four-stage displacement drum washer. The reduced POMs are regenerated, using oxygen, for the next bleaching process. Under the aggressive conditions of this step, POMs initiate and catalyze the wet air oxidation of dissolved lignin degradation compounds and other potentially present organic compounds, which are converted to carbon dioxide and water [23]. This means that the POM-based bleaching technology is designed to produce carbon dioxide and water as the only by-products. During the development of the POM bleaching technology, a number of different POM anions have been used, beginning with [PV_2_Mo_10_O_40_]^5- ^in the first reports. Further progress has been made by using [SiVW_11_O_40_]^5-^ which, in contrast to [PV_2_Mo_10_O_40_]^5-^, is stable at neutral pH levels and is characterized by a slightly higher oxidation potential. A two-step process using this POM produced delignified pulp that had properties comparable to the properties of the pulp delignifed in the CDE bleaching sequence, to the same kappa number of 4.6. New POMs, including [SiV_2_W_10_O_40_]^6-^ and [AlVW_11_O_40_]^6-^, have also been synthesized to allow reuse of POMs in numerous cycles for achieving mill closure while maintaining effective delignification [13,14,21].

The results of POM bleaching experiments on kraft pulps of different origins confirmed that POM bleaching produces pulp, which has properties that are comparable to those of the CDE pulp. The corresponding bleached pulps delignified to very low kappa number (about 4) at the same level of refining energy were of similar tensile index, burst index, tear index, and zero span tensile index. However, the CDE-bleached pulp was still superior in viscosity (viscosity of unbleached kraft pulp 28.7 mPa℘s, DE-bleached pulp 23.1 mPa℘s, POM-E bleached pulp 19.5 mPa℘s) [14]. Similar results were obtained in the comparative analysis of the properties of bleached pulps obtained in DE and POM bleaching followed by alkaline extraction (POM-E) [23]. 

An important aspect of this POM delignification concept is that the protons released during lignin oxidation in the first step are consumed by oxygen during the re-oxidation of POMs and mineralization of lignin in the second step. Taking into account this fact, the reactions 1 and 2 may be represented by the reactions 3 and 4.

2POM_ox_ + LigH_2_ → 2POM_red_ + Lig_ox_ + 2H^+^ (3)

4POM_red_ + O_2_ +4H^+ ^→ 4POM_ox_ +2H_2_O (4)

The reactions 3 and 4 clearly indicate a need for a buffer to maintain the system pH. A group of POMs described by the formula Na_5(+2)_[SiV_1(-0.1)_MoW_10(+0.1)_O_40_] has been synthesized to develop a self-buffering POM delignification system. The capability of these POMs to keep a nearly neutral pH within a small range has been demonstrated [23,24].

The two-step POM delignification process offers numerous advantages compared to other TCF bleaching technologies. These include an acceptable selectivity of POM delignification performed in a radical-free environment (anaerobic conditions), which can be improved by conducting the process in less acid conditions (by selection of a POM which is more tolerant to an increase in pH); and an effective regeneration of POMs, which provides the basis for an effluent-free technology. Unfortunately, there are some disadvantages of this approach in the application of POMs for pulp bleaching. The disadvantages are primarily related to the high concentration of POMs used in lignin oxidation; most of the delignification trials have been accomplished by using 0.5 M POM at 8–10% pulp consistency. Due to the high molar mass of POMs (the molar mass of [PV_2_Mo_10_O_40_]^5-^ and [SiVW_11_O_40_]^5-^ is 1732 g/mol and 2741 g/mol, respectively), the required quantity of POMs on a mass basis is many times higher than the quantity of agents which are conventionally used in both chorine-based (Cl_2_, ClO_2_) and chlorine-free (e.g., O_2_, O_3_, H_2_O_2_) bleaching technologies. Accordingly, to apply the two-step POM-based delignification on a commercial scale, an improvement of the process related to the efficiency of lignin oxidation with POMs is required. Elucidation of the reactions taking place during POM treatment of pulp and a better understanding of the desirable and undesirable modifications which lignin undergoes may help achieve this goal.

#### Effect on lignin

**Figure 5:**
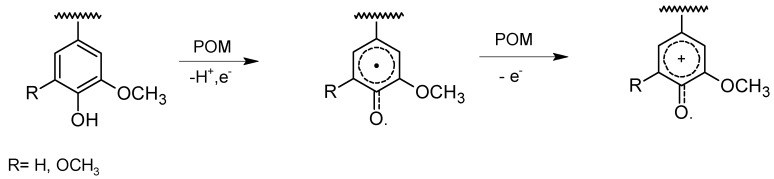
Proposed pathway for formation of the cyclohexadienyl cation upon oxidation of a phenolic lignin model compound by two equivalents of POMs [25].

The reactivity of phenolic and non-phenolic lignin structural units has been studied using monomeric and dimeric lignin model compounds (LMCs) [17,25,26,27]. A possible reaction pathway for a cleavage of the phenolic lignin model compounds has been proposed; this pathway would include two one-electron oxidation steps producing cyclohexadienyl cations from the initially formed phenoxy radicals (Figure 5) [25]. 

**Figure 6:**
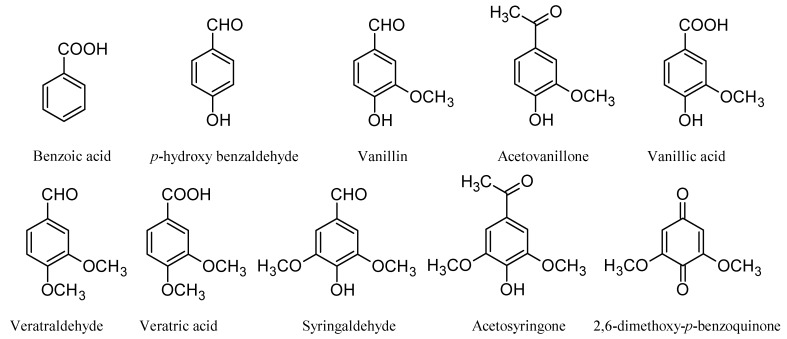
Low-molecular weight compounds identified in softwood and birch kraft pulp POM solution [32].

In contrast to phenolic LMCs, the etherified LMCs follow a different mechanism and the experiments on non-phenolic lignin subunits have revealed that the reaction may proceed via successive oxidation of the benzylic carbon atom [26]. Model studies, however, do not reflect all the reactions that lignin might undergo as a macromolecule in the pulp matrix. Oxidation of pine milled wood lignin (MWL) with POMs has also been explored [28]. The MWL was insoluble under the conditions studied, and oxidative reactions were found to be taking place primarily at the surface of the suspended lignin macromolecules, providing incomplete information on the lignin reactivity. To understand the changes which lignin undergoes during POM treatment of softwood and hardwood kraft pulps, residual lignin has been isolated from pulps at different levels of POM delignification and analyzed [29,30,31]. In addition, the nature of lignin degradation compounds dissolved during POM delignification has been explored to help elucidate lignin cleavage reactions [32]. Whereas the efforts to isolate higher-molecular weight lignin have been unsuccessful, low-molecular weight aromatic compounds have been identified in the POM spent liquor. This may be an indicator of the lignin reactions occurring on its surface; or, on the other hand, it could indicate the continuation of the degradation of higher-molecular weight lignin after dissolution, if the reactions took place in the bulk of the lignin. Among the lignin degradation products of POM treatment of different kraft pulps were acetosyringone and acetovanillone, as the products of the C_β_-C_γ_ bond cleavage; vanillin, vanillic acid, and syringaldehyde, which are the products of the C_α_-C_β_ bond cleavage; and 2,6-dimethoxy benzoquinone, which confirms the C_1_-C_α_ bond cleavage in lignin. The presence of 3,4-dimethoxy carbonyl and carboxyl aromatic compounds, veratraldehyde, and veratric acid, respectively, reveals a potential methylation reaction taking place with methanol released during the lignin demethylation reaction [28] in POM treatment of kraft pulps, since these structures are not naturally present in lignin. Benzoic acid and *p*-hydroxy benzaldehyde in the spent liquor solution may have originated from extractives remaining in the kraft pulps. The lignin origin of *p*-hydroxybenzaldehyde, however, cannot be ruled out without further investigation (Figure 6) [32]. Even though acid-catalyzed and/or homolytic cleavage of lignin in these conditions may be expected [33], common products of these processes were not identified.

**Figure 7:**
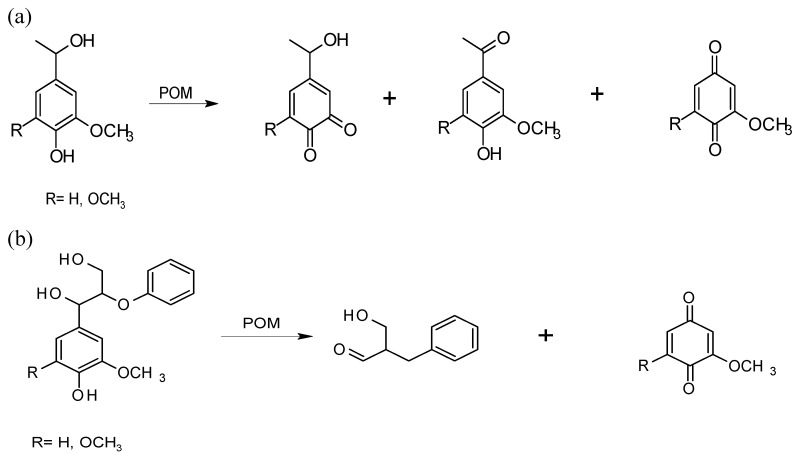
Quinonoid and aromatic products of POM treatment of phenolic LMCs: (a) monomers; and (b) dimers containing β-O-4 bond [25,27].

The results obtained using the different lignin-related substrates revealed that phenol oxidation reactions are the most significant reactions occurring during lignin oxidation with POMs. LMCs with free phenolic hydroxyl groups (PhOH) reacted both faster and at lower temperature (room temperature) than the etherified analogs, which reacted only at temperatures ≥120 °C [26]. Fast oxidation of phenolic units was confirmed in POM oxidation of kraft pulps when the content of PhOH groups rapidly decreased in the corresponding residual lignins with the progress of POM delignification [29,30,34]. Since POM oxidation of phenols yields quinones, it is expected that the quinone content in pulps will increase with the progress of POM delignification. Actually, the decrease in permanganate oxidizability observed for POM-delignified pulps, which indicates a loss of lignin aromaticity, may be an effect of quinone formation, since aromatic ring cleavage was not detected in experiments on the effect of POMs on LMCs [26,29,30,34]. The results of a number of studies on the effect of POMs on lignin have indicated quinone formation. For example, *ortho*- and *para*-quinone structures resulted from POM treatment of different lignin model compounds [25,27] (Figure 7); *para*-quinone was detected in the solution of unbleached birch kraft pulp treated with POMs [32]; a reddish-orange hue of the pulps, which is commonly noticed after treatment with POMs, was also attributed to the formation of quinones [35]. In addition, *ortho*-quinone formation in lignin is consistent with a loss of methoxyl groups (Figure 7a) [25,27,28]. Lignin demethylation has been observed in the POM treatment of pine MWL [28] and in the residual lignins of hardwood kraft pulps with the progress of POM delignification [34]. Conversely, *para*-quinone products give evidence for the occurrence of the C_1_-C_α_ cleavage reactions [25,32] (Figure 7). An abundance of quinone structures in residual lignin after POM treatment of pulp may be an important reason for a successful brightening of POM treated pulps with sodium hydroxide and hydrogen peroxide [35], as they are efficient quinone-removing agents [4]. 

In addition to quinone formation confirmed in different model studies and studies on MWL and kraft pulps, an increase in other carbonyl groups, such as -C_α_HO and -C_α_ = O, has been observed in the remaining POM-treated MWL and residual lignin of kraft pulps [28,34]. This result corroborates a reaction mechanism based on the successive oxidations of the benzylic carbon atoms**, **as proposed in the studies on etherified LMCs [26]. 

The LMC studies indicated differences in reactivity of H, G, and S lignin units (phenolic LMCs, S > G > H; etherified LMCs G > S [27,28]), whereas no difference in the delignification efficiency was observed between softwood (G lignin) and hardwood (SG lignin) kraft pulps [30]. At the beginning of the POM treatment, however, residual lignin isolated from softwood kraft pulp was of a higher PhOH content than that isolated from hardwood kraft pulp, which may have contributed to its reactivity (pulps were of approximately the same kappa number). 

Even though in kraft pulping lignin is primarily degraded through the cleavage of its most abundant β-O-4 structure (Figure 2), some β-O-4 bonds are still present in residual lignin of kraft pulps [36]. Because the bleaching result depends considerably on the agent’s ability to cleave this structure, the POM treatment of β-O-4 dimers was performed [25,27], whereby the researchers observed cleavage of the C_1_-C_α_ bond in phenolic guaiacyl- and syringyl- glycerol β-aryl ethers (Figure 7b). This finding is consistent with the observed reduction in the content of the β-O-4 bonds resulting from the POM treatment of pine MWL [28]. The 2D NMR HSQC analysis of residual lignins isolated from kraft pulps also indicated a weakening of the correlations assigned to this lignin bond [29,34]. Moreover, the products of the β-O-4 bond cleavage, identified in the LMC studies, were also identified in the POM spent liquor of the treatment of kraft pulps. This finding corroborates the delignification mechanism, which includes C_1_-C_α_ bond cleavage (Figure 6) [32]. The lignin model studies, however, did not support the claim that the delignification mechanism includes C_α_-C_β_ bond cleavage(C_α_-aldehyde and C_α_-carboxyl acid aromatic structures). Nevertheless, the cleavage of the β-O-4 bonds, the most abundant bonds in native lignin, and important bonds in residual lignin, which was revealed in the POM experiments on LMCs, MWL and kraft pulps, would contribute positively to the total delignification result in the POM treatment of kraft pulps. 

In contrast to the POM-treated pulp, unbleached kraft pulp treated under the same conditions except without POMs, showed only a minimal decrease in kappa number; the kappa numbers of unbleached mixed-pine kraft pulp, pulp treated under the same conditions without POMs, and POM-treated kraft pulp were 33.6, 29.2, and 7.4, respectively [35]. In addition to the cleavage of β-O-4 bonds, studies have suggested that the cleavage of the β-5 bond, a typical native lignin bond (Figure 2), occurred during POM treatment of pine MWL and kraft pulps [28,29,30]. The cleavage of another important lignin bond, β-β (Figure 2), was observed only during POM delignification of kraft pulps [29,30]. Accordingly, the delignification of pulps during POM treatment is attributed primarily to lignin oxidation with POMs.

**Figure 8:**
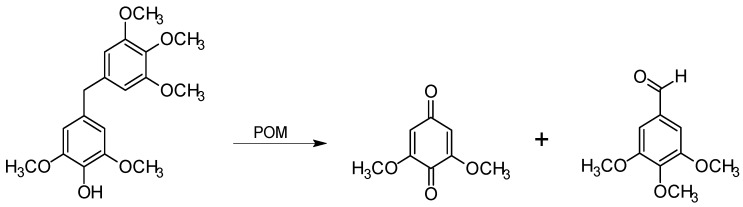
Degradation of a phenolic diphenylmethane with POMs [25].

Model studies included investigation of the phenolic diphenylmethane structure, which may appear as a result of condensation reactions in the residual lignin of kraft pulps (Figure 2) [6,8,37], although there are studies which have ruled out the existence of this condensed structure in residual lignin [38]. The C_1_-C_α_ cleavage was indicated by the presence of the *para*-benzoquinone product [25] (Figure 8). This finding is important because the relative number of C-C bonds gradually increases in residual lignin during kraft pulping and the delignification result depends on the ability of a bleaching agent to break C-C bonds in lignin. Also, an immediate disappearance of the correlations assigned to stilbene structures in the 2D NMR HSQC spectra of residual lignin of POM-treated kraft pulps indicates that stilbenes (alkali-stable structures resulting from the C_β_-Cγ bond cleavage in the β-1 and β-5 lignin structures; Figure 2) are readily modified during the POM oxidation of both softwood and hardwood (birch) kraft pulp [29,31,34].

The identification of oligomers in the model studies indicated a potential for repolymerization of lignin during the POM treatment of pulps [25]. While this undesirable path of radical coupling was confirmed in the experiments on MWL [28], no increase in polymerization degree was noticed in gel permeation chromatograms of residual lignins isolated from softwood and hardwood kraft pulps before and after POM delignification [31,34]. These conflicting results in the studies obtained using lignin models/MWL and residual lignins isolated from POM-treated pulps indicate a need for further evaluation of the efficiency of POM delignification.

Characterization of residual lignins from unbleached and POM-delignified kraft pulps showed a closer association between lignin and cellulose after the POM treatment of pulps. Alkaline extraction of residual lignin, however, successfully resulted in a decrease in carbohydrates in lignin [31]. For this reason, it is important to follow the POM treatment with an alkaline extraction step to break any potentially present alkali-labile lignin-carbohydrate associations and remove oxidized lignin fragments that are insoluble under acid conditions [14,25,31,34,35].

### 3.2. Delignification with POMs: Aerobic Conditions

POMs have been studied as catalysts in oxygen delignification of unbleached pulps in aqueous or organic solvent-water media to increase the selectivity of delignification. For this purpose, the heteropolyanions, HPA-n of the general formula [PMo_12-n_V_n_O_40_]^(3+n)-^ (n = 1–6), have been used. The most important properties of the HPA-n series of polyoxometalates are their simple re-oxidation with oxygen, a property which enables reactions (1) and (2) to occur in the same step, and their stability at pH 2.5–5, a property which requires acidic process conditions. The redox potential of the HPA-n series of POMs decreases with increasing n, and catalytic action in oxidative delignification is performed with HPA-5 in particular ([PMo_7_V_5_O_40_]^8-^, redox potential 0.60 *vs.* NHE at pH 2) [15,16,20]. Vanadium is responsible for the oxido-reduction path of the HPA-n polyoxometalates: while oxidizing lignin, HPA-5 is reduced in the reaction V^5+^ → V^4+^; reduced HPA-5 is re-oxidized with oxygen in the reaction V^4+^ → V^5+^. The VO_2_^+ ^formed via dissociation of HPA-n (reaction 5) is characterized by higher redox potential (0.87 V *vs.* NHE at pH1) than the parent HPA-n. Dissociation is more likely to occur with higher n and in strongly acidic media [16,19].

HPA-n → HPA-(n-1) + VO_2_^+^ (5)

In addition to HPA-5, the VO_2_^+ ^ions were an important contributor in catalytic oxidation of lignin. However, due to their higher redox potential, VO_2_^+ ^ions are less selective lignin oxidizing agents leading to the oxidative degradation of polysaccharides. Moreover, the free VO_2_^2+ ^ions are not oxidized by O_2_ in an acidic solution [19], which means that they cannot be recycled. Therefore, it is desirable to suppress HPA-5 dissociation, which can be done by pH control, by addition of polar organic solvents (ethanol), or by increasing the ionic strength of the solution. For example, adding ethanol into the system in the amount of up to 40–50% improves delignification selectivity due to the partial reduction of the concentration of VO_2_^+^ in the solution; greater than 60% ethanol, however, suppressed the rate of lignin oxidation due to the low concentration of VO_2_^+^ [16]. The pH of the system also strongly influences the delignification; and while degradation of polysaccharides increases with decreasing pH, the delignification efficiency decreases at pH higher than 2. 

The delignification efficiency depends on the concentration of HPA-5 and was optimal at about 2 mM, whereas at higher concentrations delignification efficiency was reduced, probably due to increase in the ionic strength in the solution [18]. To mitigate the problems related to the low selectivity of delignification and the reduction in pulp viscosity due to degradation of polysaccharides in the HPA-5/O_2_ bleaching, Mn(II)-substituted HPA-5 (HPA-5-Mn^II^) was synthesized by the use of manganese diacetate in the last step of the synthesis of HPA-5. Use of the catalyst HPA-5-1.5Mn^II^, characterized by the [HPA-5]/[Mn^2+^] ratio of 1.5, in the aerobic delignification of eucalyptus kraft pulp resulted in a slightly lower decrease in kappa number but higher viscosity of the pulp compared to HPA-5 (kappa number and viscosity of unbleached eucalyptus kraft pulp were 13.9 and 1290 cm^3^/g, respectively; HPA-5/O_2_-delignified pulp, kappa number 5.8, viscosity 920 cm^3^/g; HPA-5-1.5Mn^II^/O_2_-delignified pulp, kappa number 6.2, viscosity 1050 cm^3^/g). The higher selectivity of HPA-5-1.5Mn^II^ (designated HPA-5-Mn^II^) compared to HPA-5 may be attributed to the formation of Mn^II^ complexes with lacunary HPA-5, which is more stable than HPA-5 and produces less VO_2_^+^ in acidic conditions [39]. The feasibility of HPA-5-Mn^II^/O_2_ delignification of eucalyptus kraft pulp was confirmed in pilot-scale experiments [40]. In these experiments, a higher delignification rate was observed compared to laboratory results, but pulp viscosity was reduced more than expected (kappa number and viscosity of unbleached pulp 14.4 and 1160 cm^3^/g; kappa number and viscosity of oxygen-delignifed pulp, 7.7 and 990 cm^3^/g; kappa number and viscosity of HPA-5-Mn^II^/O_2_-delignifed pulp 6.6 and 835 cm^3^/g, respectively). Additionally, in contrast to laboratory studies in which most of the lignin/LMCs were oxidized to CO_2_, the pilot scale experiments were less successful, with the catalyst solution having a COD of 16.6 kg/ton [40,41,42]. These results showed that even though the HPA-5-Mn^II^/O_2_ delignification system represents a promising alternative for chlorine-based bleaching processes, further development and scale-up optimization of the system is necessary.

#### 3.2.1. Effect on lignin

Studies of the effect of HPA-5/O_2_ and HPA-5-Mn^II^/O_2 _on lignin have been performed using hardwood (eucalyptus) and softwood (spruce) species, monomeric and dimeric lignin model compounds, and dioxane lignin adsorbed on pulp [20,41,42,43,44]. The results of these studies indicated that the conversion of phenolic lignin units occurs 5–6 times faster than that of non-phenolic lignin units and that the syringyl units are more readily oxidized than the guaiacyl analogues. A simplified reaction scheme has been suggested by Evtuguin and Pascoal Neto [20]. They suggested that similarly to the mechanism of POM delignification in the anaerobic system, the reaction starts with one-electron oxidation of lignin phenolic units resulting in phenoxy radicals, which lose one more electron and form cyclohexadienyl cations [25] (Figure 5). In contrast to anaerobic POM treatment delignification, participation of oxygen is suggested in lignin autooxidation during aerobic delignification catalyzed by POMs/HPA-5. The role of the VO_2_^+^ released from the HPA-5 is demonstrated in the suppression of these lignin autooxidation reactions, and even more in the oxidation of non-phenolic structures for which the VO_2_^+^ ions were suggested to be the active catalysts [43]. The rate-determining step of the oxidative delignification of both phenolic and non-phenolic lignin units is the first one-electron oxidation step. Similar to the studies of anaerobic POM delignification, the studies of aerobic POM delignification indicated that the delignification includes cleavages of the C_α_-C_β_ and C_1_-C_α_ bonds, demethylation, and formation of quinone structures. The comparative analysis of lignin before and after HPA-5/O_2_ treatment of dioxane lignin adsorbed on pulp showed that the content of the β-O-4, β-β, and β-5 bonds (Figure 2) was reduced, which is the same result observed in the POM anaerobic delignification of MWL and kraft pulps [28,29,34]. In contrast to the results of POM anaerobic delignification, however, it has been suggested that during HPA-5 catalyzed aerobic oxidation of lignin in wood, aromatic ring cleavage occurs [44].

#### 3.2.2. Laccase as a catalyst of re-oxidation of POMs

As laccase may be an efficient catalyst of re-oxidation of different kinds of POMs, biocatalytic re-oxidation of reduced POMs with laccase has been proposed as a method that will enhance re-oxidation efficiency in aerobic delignification [45]. Re-oxidation can be performed in the same stage as pulp POM delignification at the temperature of ~60 °C, which is in between the temperatures required for POM delignification (90–100 °C) and for laccase activity (30–60 °C). Alternatively, laccase-catalyzed re-oxidation of POMs can be performed at a lower temperature in a separate stage after the first stage of pulp treatment with POMs at a high temperature. Experimental trials have been performed in which a two-stage process was followed by a one-stage process to achieve a higher degree of delignification with better selectivity (higher pulp viscosity). This multi-stage approach has showed the potential for further improvement [45]. 

## 4. Conclusion 

As a promising alternative to chlorine-based bleaching, oxidative delignification with polyoxometalates has been suggested. Both of the approaches, one based on the anaerobic process in which polyoxometalates are used as oxidative delignification agents, and a second based on an aerobic process in which polyoxometalates are used as catalysts, have their advantages and disadvantages. Currently, both processes require further development to make implementation feasible.
